# Versatile Use of the Small Tubular Reactor and Introduction of a Novel Design Reactor for Rapid Synthesis of Silicalite-1 Membranes

**DOI:** 10.3390/membranes16030091

**Published:** 2026-03-02

**Authors:** Rizqan Jamal, Yuta Kayukawa, Ryouki Kitamura, Manabu Miyamoto, Yasuhisa Hasegawa, Yasunori Oumi, Shigeyuki Uemiya

**Affiliations:** 1 Department of Engineering Science, Graduate School of Engineering, Gifu University, 1-1 Yanagido, Gifu 501-1193, Japan; rizqan.jamal.p7@s.gifu-u.ac.jp; 2 Department of Materials Science and Processing, Graduate School of Natural Science and Technology, Gifu University, 1-1 Yanagido, Gifu 501-1193, Japan; 3 Department of Chemistry and Biomolecular Science, Faculty of Engineering, Gifu University, 1-1 Yanagido, Gifu 501-1193, Japanuemiya.shigeyuki.j8@f.gifu-u.ac.jp (S.U.); 4 National Institute of Advanced Industrial Science and Technology, 4-2-1 Nigatake, Sendai 983-8551, Japan; yasuhisa-hasegawa@aist.go.jp; 5 Institute for Advanced Study, Gifu University, 1-1 Yanagido, Gifu 501-1193, Japan; oumi.yasunori.x2@f.gifu-u.ac.jp

**Keywords:** silicalite-1, single-gas permeance, capillary and conventional support

## Abstract

The rapid synthesis of high-performance silicalite-1 membranes was systematically investigated by examining the effects of seed size, solution volume, and reactor configuration on membrane growth, microstructure, and gas separation performance. Silicalite-1 seeds (~100 nm and ~1 µm) were dip-coated onto capillary α-alumina supports, followed by secondary growth under controlled conditions. Small seeds (~100 nm) produced high nucleation density, uniform intergrowth, and defect-free membranes, yielding consistently high ideal separation factor for H_2_/SF_6_ (181–295) and low SF_6_ permeance (~10^−9^ mol m^−2^ s^−1^ Pa^−1^) after only 45 min of synthesis. In contrast, larger seeds (~1 µm) enabled faster growth but resulted in less uniform layers with inferior selectivity. Furthermore, a novel reactor design with enhanced heat transfer enabled the rapid silicalite-1 membrane synthesis on conventional large-diameter tubular supports, producing well-intergrown and uniform membranes with high H_2_ permeance (4.7 × 10^−6^ mol m^−2^ s^−1^ Pa^−1^) and high ideal separation factors of up to 349 for H_2_/SF_6_ and 223 for N_2_/SF_6_. Overall, this study demonstrates that optimization of seed properties, synthesis parameters, and reactor design enables rapid and scalable fabrication of silicalite-1 membranes with robust molecular sieving performance, highlighting their strong potential for SF_6_ purification applications.

## 1. Introduction

Zeolite membranes have attracted considerable attention for their potential in molecular separation. Zeolites are crystalline aluminosilicates with a well-defined pore structure, high thermal stability, and chemical resistance. These properties make them promising candidates for gas separation [1,2,3], pervaporation [4,5,6], and membrane reactors [7,8,9]. A key advantage of zeolite membranes lies in their uniform pore size, which enables molecular sieving and allows the selective transport of molecules based on size and shape. Furthermore, their hydrophilic or hydrophobic nature can be tailored by adjusting the Si/Al ratio, making them highly versatile for separating water from organic compounds [10,11,12,13] or for gas dehydration [14,15].

Despite their potential, zeolite membranes face challenges related to high cost and long synthesis times [16]. Most of the synthesis time is consumed by hydrothermal synthesis under high temperature and pressure, resulting in significant energy consumption. Therefore, reducing synthesis time without sacrificing membrane quality has become a critical objective for advancing both laboratory research and industrial application of zeolite membranes.

Although microwave-assisted methods can greatly shorten the synthesis duration [17,18,19], their industrial scalability remains limited. In 2016, a rapid hydrothermal method using a tubular reactor reduced the synthesis time for crystalline MFI zeolite powder from days to 10 min, later to 5 min [20,21]. Recently, Li et al. optimized the synthesis of *b*-oriented platelike MFI crystals with 15 min crystallization time, which was enabled by engineering a tubular reactor with a small and thin pipe wall [22]. For membrane synthesis, Qiu et al. synthesized silicalite-1 membranes (silicalite-1 is a pure silica form of MFI) using ammonium hexafluorosilicate in a fluoride-mediated system at 80–100 °C within 3–12 h [23]. Similar acceleration using a tubular reactor in the synthesis of zeolite crystals was achieved for SAPO-34 (CHA-type silicoaluminophosphate) and SSZ-13 (high-silica CHA-type aluminosilicate) membranes [24,25], indicating broader applicability of the strategy proposed by Li et al [22].

Microwave-assisted membrane growth showed promising results [26], but these methods still require longer times than MFI powders and face scale-up limitations [27]. To address these challenges, we previously reported the rapid fabrication of CHA membranes [4]. Uniform heating and rapid temperature elevation in a small tubular reactor (inner diameter, ID = 4.0 mm; outer diameter, OD = 6.0 mm; length, L = 135 mm), combined with the use of α-Al_2_O_3_ capillary supports (OD = 2.5 mm; ID = 2.0 mm), enabled successful synthesis of CHA membranes with high separation performance in only 30 min. This reactor design, characterized by a high surface-to-volume ratio and short heat transfer distances, is key to enabling such an unprecedented reduction in synthesis time.

MFI-type zeolite—including ZSM-5 (aluminosilicate), silicalite-1 (pure silica form), and TS-1 (titanosilicate), which shares the same MFI framework topology [28]—is one of the most extensively studied zeolites and is widely used in industry. Its hydrophilicity or hydrophobicity can be tuned by adjusting the framework composition [29,30]. MFI topology contains two types of 10-membered ring channels: a straight channel (0.56 × 0.54 nm) and a zigzag channel (0.55 × 0.51 nm) [31,32]. This pore system is considered suitable for SF_6_ separation, which is used mainly in gas-insulated switchgear units [33], because the kinetic diameter of SF_6_ (~0.56 nm) [34] is larger than that of N_2_ (~0.365 nm) [35]. Zhang et al. recently reported that silicalite-1 (pure silica MFI) membrane exhibited higher N_2_/SF_6_ separation efficiency than SAPO-34 membranes, which have smaller pore systems, due to high N_2_ permeance, although the N_2_/SF_6_ selectivity of the SAPO-34 membrane was much higher than that of the silicalite-1 membrane [1].

**Figure 1:**
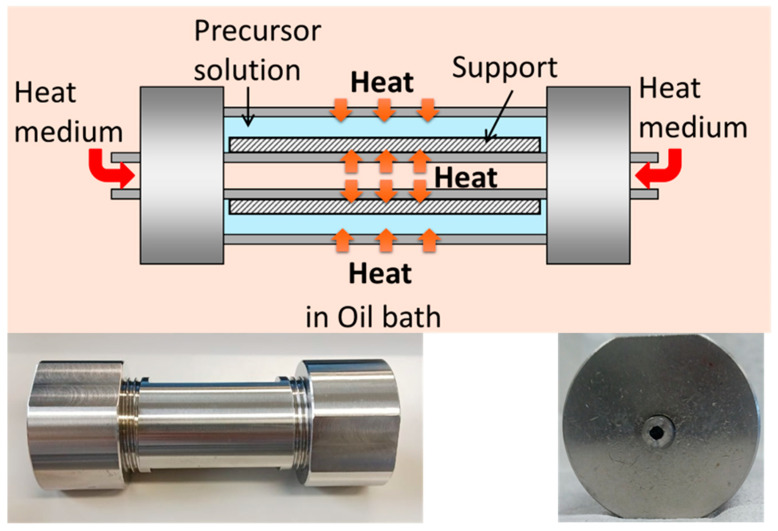
Heat transfer-enhanced reactor for conventional large-diameter support.

In this study, we investigated the rapid synthesis of silicalite-1 membranes to demonstrate the versatility of our approach and its potential for scale-up. Hydrothermal synthesis was performed using a small tubular reactor (ID = 4.0 mm, OD = 6.0 mm, L = 100 mm) for capillary supports, similar to that reported previously [4] but with a reduced length, and a newly designed reactor with improved heat transfer for conventional large-diameter supports (OD = 10 mm, ID = 7.0 mm) as shown in Figure 1. The main body of the reactor has an outer diameter of 30 mm, an inner diameter of 14 mm, and a length of 80 mm, providing an aspect ratio (*L*/*D*) of 5.7. The outer and inner diameters of the inner tube are 6 mm and 4 mm, respectively. The conventional support used in this study had a length of 3 cm. This corresponds to a surface area of 18.8 cm^2^, which is larger than that of the capillary support (5.1 cm^2^) with the same effective length of 3 cm. By maintaining rapid and uniform heat transfer even for larger supports, this reactor design directly addresses a key bottleneck in scaling up rapid zeolite membrane synthesis. We designed the new reactor for conventional large-diameter support to facilitate enhanced heat transfer, with the following factors being considered:

i. Reducing the reactor size, which is primarily important to improve heat transfer.

ii. Reducing the inner volume of the reactor by an inner tube, which can reduce the effective synthesis solution volume.

iii. Assisting heat transfer from the reactor inside by the heat transfer medium circulating through the inner tube.

To minimize membrane defects during the very short synthesis time, the effect of seed crystal size was investigated using capillary supports. Then, the scalability of the rapid membrane synthesis was demonstrated using conventional large-diameter supports. This work represents a significant step toward optimizing zeolite membrane fabrication and improving the productivity of hydrothermal synthesis.

## 2. Experiment

### 2.1. Materials

Colloidal silica (LUDOX^®^ AS-40, 40 wt%, Sigma-Aldrich, St. Louis, MO, USA; and 30 wt% suspension, JGC Catalysts and Chemicals Ltd., Tokyo, Japan), tetra-*n*-propylammonium bromide (TPABr, 98%, Nacalai Tesque, Inc., Kyoto, Japan), tetra-*n*-propylammonium hydroxide (TPAOH, 10 wt%, Tokyo Chemical Industry Co., Ltd., Tokyo, Japan), tetraethyl orthosilicate (TEOS, 95%, FUJIFILM Wako Pure Chemical Corporation, Osaka, Japan), sodium chloride (99.5%, Nacalai Tesque, Inc., Kyoto, Japan), sodium hydroxide (97%, Nacalai Tesque, Inc., Kyoto, Japan), ammonia solution (28 wt%, Nacalai Tesque, Inc., Kyoto, Japan), and nitric acid (67 wt%, Tokyo Chemical Industry Co., Ltd., Tokyo, Japan) were used without any pretreatment. Homemade α-alumina capillary supports (outer diameter, OD = 2.5 mm, inner diameter, ID = 2.0 mm, average pore diameter (APD) = 150 nm) and conventional large-diameter α-alumina supports (OD = 10 mm, ID = 7.0 mm, APD = 150 nm) were used.

### 2.2. Synthesis of Silicalite-1 Seed Crystal

Small silicalite-1 seed crystals were prepared based on a previously reported method [36]. A 20 wt% aqueous solution of TPAOH (20 g) was mixed with TEOS to obtain a solution with the molar ratio of TEOS:TPAOH:H_2_O = 1.0:3.7:4.7. The mixture was stirred at room temperature overnight and then heated at 60 °C to remove water and alcohol. The resulting concentrated solution was transferred into a PTFE-lined stainless-steel autoclave and crystallized by hydrothermal treatment at 170 °C for 48 h. The solid product was recovered by centrifugation, washed several times with distilled water, and dried overnight at 80 °C.

Large silicalite-1 seed crystals were synthesized hydrothermally using a gel with the composition of SiO_2_:TPABr:NaOH:H_2_O = 1.0:0.7:0.5:100. TPABr was first dissolved in 133 g of distilled water in a 1 L PTFE beaker under stirring at room temperature. Colloidal silica (30 wt% suspension) was then added gradually to the TPABr solution while stirring until a homogeneous mixture was obtained. In a separate beaker, sodium hydroxide was dissolved in 34 g of distilled water to form a clear solution, which was subsequently introduced dropwise into the silica–TPABr mixture under continuous stirring. The resulting synthesis gel was stirred for 10 min at room temperature, maintained at 60 °C under vigorous stirring for 24 h, and then heated at 100 °C under stirring for 4 days. The solid product was collected by filtration, washed several times with distilled water.

Both small and large seed crystals were calcined in air at 823 K for 10 h to remove the organic structure-directing agent (OSDA) before seeding.

### 2.3. Synthesis of Silicalite-1 Membrane

A silicalite-1 membrane was synthesized using a precursor solution with the molar composition SiO_2_:TPABr:TPAOH:NaCl:H_2_O = 1:0.05:0.05:0.05:20. The solution was stirred for 1 h at room temperature in a PTFE container. For capillary supports, seeded supports were prepared using the dip-coating method, in which the support was withdrawn from the 1 wt% and pH = 8 seed crystal solution at a rate of 5 mm/min and then dried overnight at 80 °C; this process was repeated twice. For conventional large-diameter supports, the rubbing method was employed, where seed crystals were rubbed onto the support for 5–10 min, followed by drying overnight at 80 °C. The seeded supports were then calcined at 500 °C for 2 h, which was applied to enhance seed adhesion to the support surface and reduce seed detachment during hydrothermal synthesis. For secondary growth, the precursor solution was loaded into the reactor with the seeded support and then placed horizontally in an oil bath at 190 °C for various synthesis durations, where the capillary support positioning was not fixed, but both ends of the support were capped by PTFE tape to prevent the support surface from contacting the reactor wall. After hydrothermal synthesis, the reactor was rapidly cooled with tap water, and the membrane was removed, lightly washed in an 80 °C water bath, thoroughly rinsed, and further washed for 30 min in hot water five times. The membrane was then dried overnight at 80 °C and calcined at 500 °C for 12 h using a controlled heating and cooling rate of 1 °C/min to remove OSDA and activate silicalite-1 membranes for gas permeation tests.

### 2.4. Characterization

X-ray diffraction patterns were collected using an X-ray diffractometer (XRD D8 ADVANCE, Bruker, Billerica, MA, USA) with Cu Kα (1.54060 Å) at 40.0 kV and 40.0 mA. The obtained patterns were compared with the standard reference pattern from the International Zeolite Association database [37]. The morphology of seed crystals and membranes was observed using a high-resolution field-emission scanning electron microscope (Hitachi High-Technologies, S-4800, Tokyo, Japan). The elemental composition of the membrane was determined by energy-dispersive X-ray spectroscopy (EDX, Oxford Instruments, Abingdon, Oxfordshire, UK). The chemical composition of the prepared seed crystal was analyzed by wavelength-dispersive X-ray fluorescence analysis (XRF S8 TIGER, Bruker, Billerica, MA, USA).

### 2.5. Gas Separation

The gas separation performance of the membranes was evaluated through single-gas permeation experiments using hydrogen (H_2_), nitrogen (N_2_), and sulfur hexafluoride (SF_6_) at room temperature and Δ*P* = 0.15 MPa. The feed flow rate was maintained between 20 and 100 mL (STP)/min, with higher flow rates required for membranes on conventional supports. The permeate side was kept at atmospheric pressure. After reaching steady-state conditions, the permeate flow rate was measured using a soap bubble flowmeter. The effective membrane area was approximately 150 mm^2^ for capillary supports and 600 mm^2^ for conventional supports. The gas permeance *P_i_* [mol·m^−2^·s^−1^·Pa^−1^] was calculated according to Equation (1):

*P_i_* = *N_i_*/(*A* × Δ*p_i_*) (1)

where *N_i_* is the molar flow rate of the permeate gas *i*, *A* is the membrane area, and Δ*p_i_* is the partial pressure difference of gas *i* across the membrane.

The ideal separation factor *α_i/j_* was determined using Equation (2):

*α_i_*⧸*_j_* = *P_i_*/*P_j_* (2)

This parameter reflects the membrane’s selectivity for gas *i* over gas *j*.

## 3. Results and Discussion

### 3.1. Rapid Synthesis of Silicalite-1 Membrane on Capillary Supports

We have previously demonstrated that the strong attachment of seed crystals to the support surface is a key for successful secondary growth, as these seeds play a crucial role in zeolite membrane synthesis, particularly under rapid synthesis conditions [4]. In this section, the primary objective of manipulating seed crystal size is to achieve an optimal seed distribution, since our earlier work showed that support with fewer crystal-void regions and minimal seed aggregation promoted superior membrane growth.

**Figure 2:**
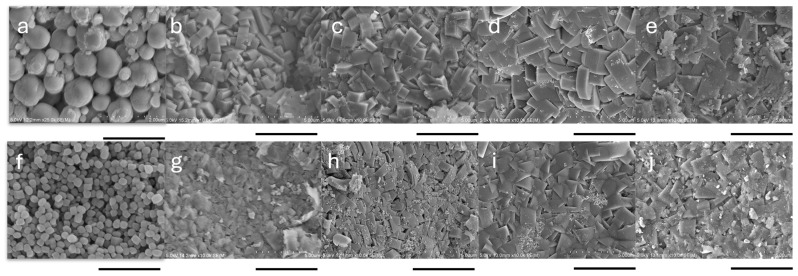
SEM image of the surface of silicalite-1 membranes synthesized using L-seed (**a**–**e**) and S-seed (**f**–**j**) on the capillary support for 0 (seeded support), 15, 30, 45, and 60 min, respectively. Scale bar: 2 µm for seeded supports and 5 µm for membranes.

Two types of silicalite-1 seed crystals with different sizes were prepared: large seeds (L-seed) with an average size of approximately 1 µm, and small seeds (S-seed) with an average size of around 100 nm, as shown in Figure S1. The XRD patterns of both seed crystals, presented in Figure S2, showed a prominent (501) peak for both L-seed and S-seed crystals, while the (101) peak was more intense for L-seed than S-seed. Furthermore, XRF analysis confirmed that each seed crystal contained 99.99% silica. Figure 2a,f shows SEM images of seeded supports with L-seed and S-seed. In both cases, the seeded layers successfully covered most of the support surface. Nevertheless, the L-seed-coated support exhibited more uncovered regions and less uniform coverage, particularly in the uppermost seed layer. In contrast, these nonuniformities were significantly reduced or largely avoided in the S-seed-coated support.

**Figure 3:**
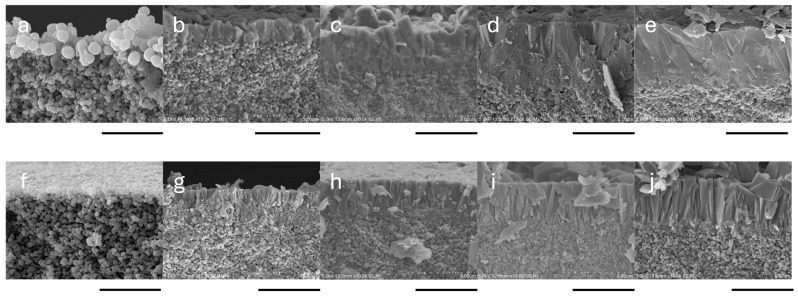
SEM image of the cross-section of silicalite-1 membranes synthesized using L-seed (**a**–**e**) and S-seed (**f**–**j**) on the capillary support for 0 (seeded support), 15, 30, 45, and 60 min, respectively. Scale bar: 5 µm.

Silicalite-1 membranes were successfully synthesized using seeded supports prepared with both L-seed and S-seed, as confirmed by Figure 2 and Figure 3. SEM images of the membrane cross-section and surface reveal successful secondary growth during hydrothermal synthesis in the small tubular reactor, even within very short durations. The rapid secondary growth of silicalite-1 is attributed to enhanced heat transfer provided by the small tubular reactor, as demonstrated in CHA membranes [4]. While multiple calcination steps were applied in this study to ensure reliable seed crystallinity, adhesion, and membrane activation, their use may also influence synthesis efficiency and scalability. From a process optimization perspective, the first and second calcination steps are not essential and could be simplified or eliminated without compromising membrane quality. This highlights an opportunity to further improve synthesis productivity, which will be systematically investigated in future work.

Cross-sectional SEM images of silicalite-1 membranes synthesized using L-seed and S-seed crystals revealed clear differences in both membrane thickness and microstructural development. For the L-seed membranes, the thickness increased steadily from approximately 1.7 µm, 2.1 µm, and 3.5 µm to 4.0 µm at crystallization times of 15, 30, 45, and 60 min, respectively. In comparison, the S-seed membranes were consistently thinner at the same synthesis time, with measured thicknesses of 1.0 µm, 2.0 µm, 2.9 µm, and 3.6 µm under the same synthesis conditions. These results indicate that the L-seed crystals facilitated faster and more extensive growth of the silicalite-1 layer, resulting in thicker membranes.

The observed increase in surface crystal (grain) size with longer synthesis time is consistent with general crystallization behavior under secondary growth, as shown in Figure 3, and underscores the important role of seed size and seeding conditions in controlling membrane microstructure. As shown, L-seed-derived membranes developed larger surface crystals. Previous studies have also reported that seed crystal size significantly influences zeolite membrane synthesis. Specifically, smaller seeds (approximately 100 nm) tend to form uniform and dense seed layers, while larger seeds (600 nm–3 μm) produce coarser structures with poorer intergrowth [38]. A comprehensive study on MFI-type membranes noted that small seeds (100–200 nm) yield better intergrowth, more uniform layers, and often better *c*-orientation with extended synthesis, whereas larger seeds (1–2 μm) tend to produce coarser and less well-intergrown films [39]. These findings are consistent with our study: L-seed membranes exhibited larger surface crystals and coarser morphology relative to S-seed membranes under the same synthesis conditions. Moreover, the interplay between seed size and growth kinetics might influence membrane orientation and layer continuity. As reported in the literature, small seeds favor oriented and dense membranes with fewer non-zeolitic pathways under extended synthesis time [39]. In contrast, large seeds may grow in various directions due to lower steric constraints, leading to more random orientation and coarser grain boundaries. As the membrane grew in different directions, this resulted in a more defective zeolite layer [40]. Therefore, although L-seed membranes may exhibit larger crystals with time, these structural characteristics may compromise membrane performance, particularly selectivity.

Furthermore, the formation of larger crystals with increasing synthesis time likely results from continued growth and coalescence of zeolite units once nucleation sites are established. This observation aligns with previous reports; for example, in a study on ZSM-5 crystals grown from silicalite-1 seeds, crystal size increased gradually with synthesis time and reached a plateau after several hours [41]. Similarly, Du et al. reported that in CHA zeolite membranes, both crystallinity and membrane thickness increased with synthesis time, indicating ongoing crystal growth [42]. Thus, our observations during the short synthesis period of 15–60 min resemble the common growth trajectory observed in the conventional long-duration synthesis: after initial nucleation, crystal growth proceeds over time, enlarging individual crystals on the membrane surface. While our previous report on the CHA membrane indeed showed that the crystal edge became more prominent as the synthesis time increased, there was not much difference in membrane thickness [4].

In our previous work, the synthesized CHA membrane exhibited a thin zeolite layer of approximately 0.5 µm, which we suspected was due to the limited volume of the synthesis solution. To investigate whether increasing the solution amount could enhance membrane growth, thickness, and performance, the synthesis solution volume was doubled by extending the reactor length from 100 mm to 200 mm. However, cross-sectional SEM images (Figure S3a) showed no significant change in silicalite-1 membrane thickness, and surface SEM images (Figure S3b) revealed no notable morphological differences between membranes prepared using the two reactor lengths. Under the current synthesis conditions, membrane thickness continues to increase beyond 60 min, indicating that unlike the CHA case, not all raw materials in the reactor are consumed within this time frame. Consequently, increasing the synthesis solution volume did not lead to clear changes in membrane thickness or morphology, suggesting that reactor length and solution volume have minimal morphological influence under the synthesis conditions used for silicaite-1 membrane in this study.

**Figure 4:**
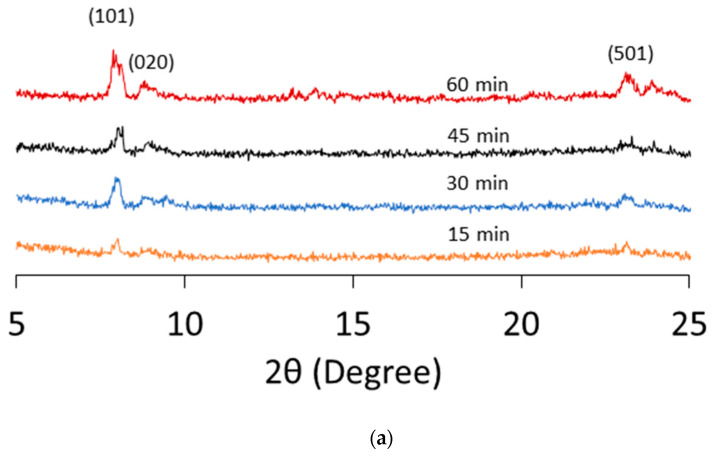

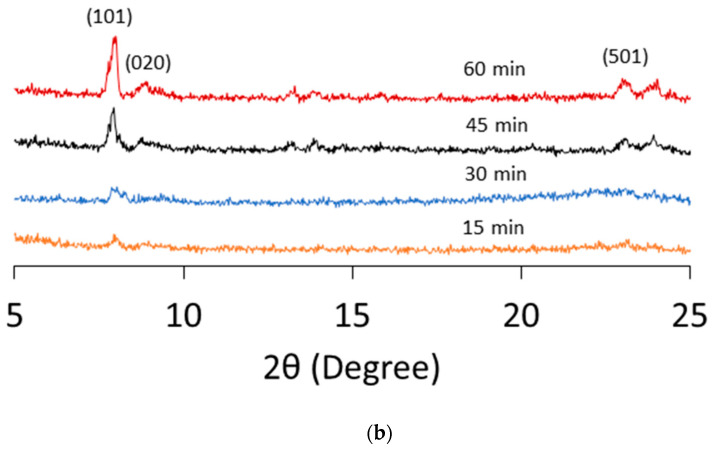
XRD patterns of time variation of the silicalite-1 membranes prepared on the capillary supports with (**a**) L-seed and (**b**) S-seed.

The X-ray diffraction (XRD) patterns of the silicalite-1 membranes are shown in Figure 4. Distinct Bragg peaks were observed at 2θ values of 7.9° (101), 8.9° (020), and 23.1° (501), which are characteristic of the MFI-type zeolite structure [43]. For the L-seed membrane, the XRD pattern showed an increase in peak intensity with membrane growth time, with the (101) peaks exhibiting the highest intensity. The S-seed membrane, synthesized for 60 min, followed the same trend as the L-seed; its peak intensities increased compared to the 45 min sample. These XRD observations are consistent with the SEM observation, which showed increased membrane thickness with extended synthesis time. In XRD patterns of seed crystals, as shown in Figure S2, S-seed showed a lower intensity of peak (101) than L-seed, while a similar intensity was observed at the (501) peak.

Zhang et al. reported that the peak intensity ratio of (101) over (020) (denoted as *I*(101)/*I*(020)) improved rapidly from 0.6 in 10 h to 3.6 after 24 h. The relatively higher intensity of the (101) peak than that of the (020) peak on the membrane obtained after 24 h reflected that MFI crystals grew with their [*h*0*h*] orientation on the membrane surface, which could be further revealed by the surface of the SEM photograph [44]. In this study, *I*(101)/*I*(020) of L-seed and S-seed membranes exhibited 1.38 and 1.14, respectively, even in 15 min synthesis, and were increased to 1.97 and 1.32 in 60 min synthesis. This indicates preferential membrane growth in the *c*-direction of the silicalite-1 membrane in very short time synthesis.

Energy-dispersive X-ray spectroscopy (EDX) mapping was carried out on both the surface and cross-sectional regions of the silicalite-1 membranes synthesized using L-seed and S-seed crystals to examine elemental distribution and interfacial characteristics, as shown in the Supplementary Materials (Figure S4). The EDX elemental maps revealed that the membrane region was predominantly composed of silicon (Si), represented in red, confirming the formation of a continuous silicalite-1 layer. In contrast, aluminum (Al), shown in green, was only detected in the cross-sectional view beneath the membrane, corresponding to the underlying alumina support. Notably, the L-seed membranes, a distinct composite layer, were clearly visible beneath the silicalite-1 membrane. This configuration exhibited a greater degree of Si penetration into the alumina support compared to the S-seed membranes, suggesting the formation of a thicker composite interfacial region. However, this deeper Si incorporation may have increased diffusion resistance, which could explain the poorer separation performance observed for the L-seed membranes relative to the S-seed membranes, as discussed later.

**Figure 5:**
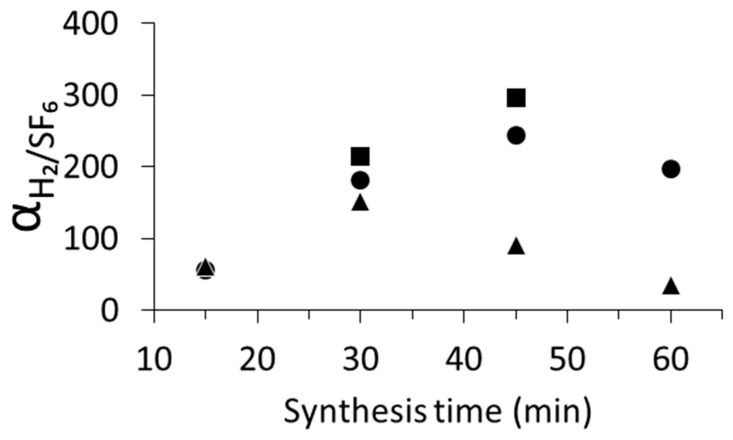
Ideal separation factor of H_2_ over SF_6_ with synthesis time for silicialte-1 membranes on the capillary supports using L-seed (▲) and S-seed (●) in 10 cm reactor and S-seed in 20 cm reactor (■).

The single-gas permeation performance of silicalite-1 membranes is investigated and summarized in Figure 5. SF_6_, whose kinetic diameter is comparable to the pore sizes of MFI, has frequently been used as a proof molecule to assess membrane defects, although it can still permeate through MFI channels at a very low rate [45]. In addition, SF_6_ is widely used in a variety of industrial processes but is recognized as one of the most potent greenhouse gases [46].

The separation performance of silicalite-1 membranes is governed by the intrinsic diffusivity of molecules within the pores and the presence of non-zeolitic pathways (defects). The ideal separation factor, α_H2/SF6_, is highly sensitive to defects in silicalite-1 membranes. In our experiments, α_H2/SF6_ showed a strong dependence on seed size (i.e., seed crystal distribution and adhesion to the support during synthesis) and on synthesis time. For L-seed membranes, α_H2/SF6_ initially increased from 60 at 15 min to 152 at 30 min. Although the membrane thickness increased by only 23% during this period, this significant improvement in α_H2/SF6_ indicates the secondary growth covered much of the remaining intercristal voids between the seeds.

Nevertheless, extending the synthesis time beyond 30 min led to a sharp decline in α_H2/SF6_ to 35 at 60 min on the L-seed membrane. Prolonged crystallization is known to promote excessive crystal growth, resulting in larger membrane crystals [47,48], and the increased thickness, as shown in Figure 3, was consistent with that. However, importantly, the relationship between membrane thickness and defect density is not straightforward. While thinner layers may be formed, smaller crystals can introduce a higher density of grain boundaries and associated defects [24,47]. Conversely, increasing membrane thickness does not necessarily suppress defects, as additional crystal growth may induce stress and cracking within an already continuous layer [47,49]. Similar trends have been reported by Du et al., who observed a decrease in separation factor when the synthesis time was extended from 48 to 72 h, which was mainly attributed to the overgrowth of membrane crystals resulting in large intercrystalline boundary defects [42].

In contrast, silicalite-1 membranes synthesized using S-seed consistently exhibited higher α_H2/SF6_: the 15 min sample showed 56, which dramatically increased to 181 at 30 min; further growth to 45–60 min yielded high α_H2/SF6_ of 244 and 201, respectively, indicating the formation of a highly selective, near-defect-free membrane in a very short time. SF_6_ permeance for the 45 and 60 min membranes remained very low (<3 × 10^−9^ mol m^−2^ s^−1^ Pa^−1^), confirming minimal non-selective transport.

The literature reported that seed crystal size strongly affects permeance, with some studies directly stating its influence on SF^6^ permeance and the resulting H_2_/SF_6_ ideal separation factor. For example, MFI membranes prepared with small seeds (100–200 nm) showed SF_6_ permeance roughly three times lower than those prepared with large seeds (1–2 µm), resulting in H_2_/SF_6_ ideal separation factor well over 100 after full growth (~6 h) [39]. Other reports explained the influence of seed size on the membrane morphology. When spherical seed crystals were used, the membrane surface consisted of randomly oriented crystals [50], and randomly oriented polycrystalline MFI membranes were obtained with larger seeds (1–2 µm) [39]. Sakai et al. reported that silicalite-1 membrane composed of fewer grain boundaries, prepared using small seed crystals (268 nm), retained the original MFI pore size. In contrast, the membrane prepared with larger seed crystals (1 µm) contained many grain boundaries, resulting in a relatively small micropore volume, and reduced effective pore size was observed [51]. S-seed membranes consistently exhibited higher α_H2/SF6_ than L-seed membranes. It is concluded that S-seed seeding combined with controlled secondary growth promoted superior membrane quality with fewer grain boundaries than membrane produced with L-seed. Under rapid synthesis, S-seed provides more favorable conditions for secondary growth, in agreement with previous reports.

Figure S5 shows the relationship between α_H2/SF6_ and the peak intensity ratio of silicalite-1 over Al_2_O_3_ support on the S-seed membranes; this helps us to understand the membrane growth mechanism. In the initial 30 min, the α_H2/SF6_ was significantly improved from 15 min to 30 min as the silicalite-1/Al_2_O_3_ peak ratio increased. However, the α_H2/SF6_ improvement was likely to be less pronounced compared with the increase in the peak ratio beyond 30 min. This suggests that most intercrystalline voids between seed crystals disappeared during the initial 30 min and that further prolonged synthesis mainly promoted the membrane growth in the membrane thickness direction and had a relatively minor effect on healing the membrane defects.

The gas permeation performance of the silicalite-1 membrane synthesized in the 20 cm reactor was also evaluated. The silicalite-1 membranes prepared using S-seed in the long reactor showed an improvement in α_H2/SF6_ of 213 compared to 181 for the 10 cm reactor after 30 min of synthesis, while H_2_ permeance remained at the same order of magnitude (5.4 × 10^−7^ mol m^−2^ s^−1^ Pa^−1^). This indicates that although membrane thickness and continuity were similar, the longer reactor reduced defect density. Increasing the synthesis time to 45 min resulted in a more consistent and generally higher separation performance. Notably, the highest separation factor reached 296. This enhancement can be attributed to reduced defects, which favor the transport of smaller H_2_ molecules while restricting larger SF_6_ molecules.

### 3.2. Rapid Preparation of Silicalite-1 Membrane on Conventional Large-Diameter Support

**Figure 6:**
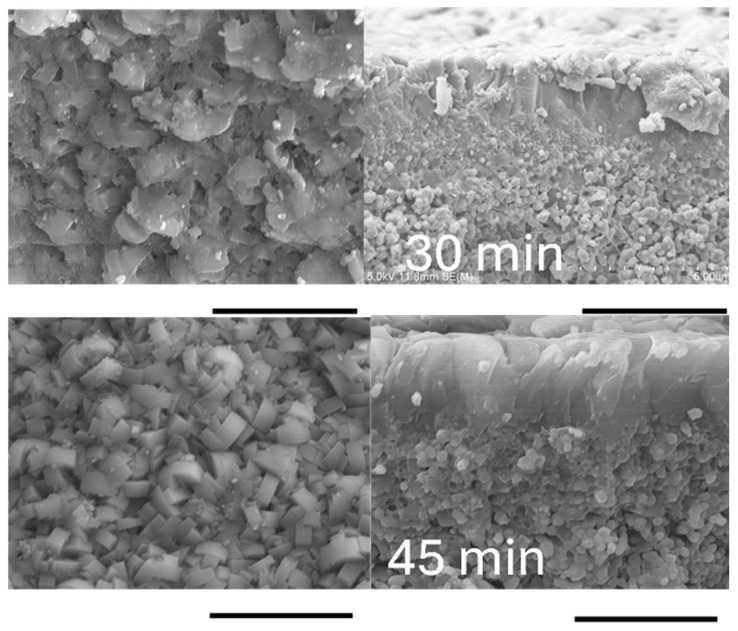
SEM image of silicalite-1 membranes synthesized for 30 and 45 min using S-seed on conventional large-diameter support. Scale bar: 5 µm.

We successfully prepared silicalite-1 membranes on a conventional large-diameter support using the secondary growth method. After rubbing S-seed onto the support (Figure S6 in the Supplementary Materials), the seeded support was calcined prior to hydrothermal synthesis, following a procedure similar to that used for dip-coated capillary supports. The membrane surface exhibited a rectangular morphology, with the 45 min membrane showing a more well-defined shape, as shown in Figure 6. Cross-sectional images also revealed a distinct silicalite-1 membrane layer on the support, which appeared more pronounced in the 45 min membrane. The membrane thickness was 2.7 µm and 3.6 µm in 30 min and 45 min synthesis, respectively, comparable to those using the capillary supports. In addition, EDX mapping confirmed the presence of the silicalite-1 layer on the support with minor Si penetration as presented in Figure S7. For the 30 min membrane, a composite interfacial layer was observed between the zeolite layer and the support (Figure 6); the combined thickness of this composite layer and the zeolite layer was approximately 3.6 µm. This observation provides insight into the silicalite-1 membrane growth sequence in this study, suggesting that membrane formation initiates at the outer surface of seeded support through interaction with the precursor solution, followed by inward growth and gradual development of the composite seed layer into a continuous zeolite layer with extended synthesis time. It is also possible that the precursor solution contacted the composite layer originating from the inner surface of the support. Given that the support inner diameter is 7 mm and the inner tube diameter is 6 mm, the resulting gap may have allowed a portion of the precursor solution to permeate through the support pores. With sufficient synthesis time, this could have initiated secondary growth from the inner side, leading to the formation of the 45 min membrane with a reduced composite layer.

**Figure 7:**
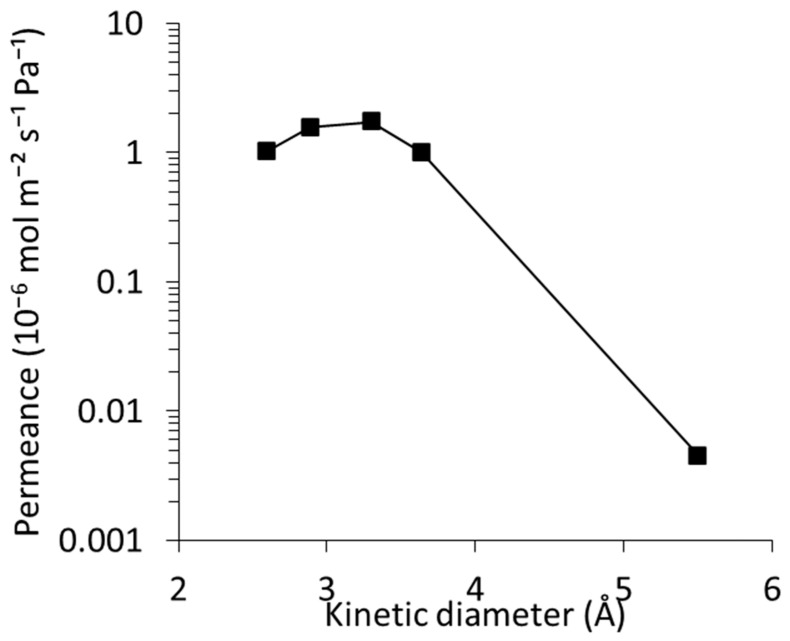
Gas permeance as a function of kinetic diameter for the 45 min membrane synthesized on a conventional large-diameter support. Kinetic diameters of the gases are He (2.6 Å), H_2_ (2.9 Å), CO_2_ (3.3 Å), N_2_ (3.6 Å), and SF_6_ (5.5 Å).

The single-gas permeation performance of silicalite-1 membranes synthesized on the conventional supports, along with a comparison to the membranes prepared on the capillary supports, is presented in Table 1 and in Figure 7. The 30 min membranes exhibited selectivity, but α_H2/SF6_ was relatively lower compared to that on the capillary supports. Interestingly, the silicalite-1/Al_2_O_3_ peak ratio and the separation factor of the 30 min membrane on the conventional support were 0.26 and 56, respectively, which were comparable to those of the 15 min membrane on the capillary support, although the membrane thickness was greater (2.7 μm for conventional support in 30 min and 1.0 μm for capillary support in 15 min). This suggests a time delay in crystallization inside the silicalite-1 polycrystalline layer in the case of the conventional supports, while the pseudo-dense silicalite-1 layer was formed in the first 30 min on the conventional support, as in the case of the capillary support. Although the reactor used for the conventional supports is sufficiently small compared to a conventional reactor, it is still larger than those used for the capillary supports. Consequently, the reactor has a larger heat capacity than the small tubular reactor, and the temperature rise inside the reactor during the initial stages of synthesis would be smaller. This likely resulted in relatively less crystallinity of the silicalite-1 layer, leading to lower α_H2/SF6_ at the initial synthesis stage. Indeed, in the 30 min synthesis, the silicalite-1 membrane on the capillary support exhibited sharper crystal edges on the membrane surface (Figure 2) compared to membranes prepared on the conventional support (Figure 6), suggesting more advanced secondary growth. In contrast, the 45 min membranes exhibited high α_H2/SF6_ in the range of 261 ± 165, comparable to those of the membranes on the capillary supports. In addition, the silicalite-1/Al_2_O_3_ peak ratio in the 45 min membranes was also very similar regardless of the support types. This implies the difference in the membrane growth between the capillary and conventional supports is in the first 30 min synthesis and less beyond 30 min, where most intercrystalline voids disappeared. Furthermore, the H_2_ permeance of membranes on the conventional supports was found to be one order of magnitude higher than that of membranes on the capillary supports owing to the larger porosity of the conventional support.

Figure 7 shows the single-gas permeance of silicalite-1 membrane on the conventional support with the best α_H2/SF6_. The membrane exhibited the following permeance sequence: CO_2_ (1.7 × 10^−6^) > H_2_ (1.6 × 10^−6^) > He (1.0 × 10^−6^) ≈ N_2_ (1.0 × 10^−6^) >> SF_6_ (4.5 × 10^−9^ mol m^−2^ s^−1^ Pa^−1^) with an H_2_/SF_6_ ideal separation factor of 349. This confirms negligible macroscopic defects and suggests that molecular transport occurs almost exclusively through zeolitic channels. CO_2_ permeated more rapidly than expected from size alone, consistent with quadrupole-induced adsorption enhancement within MFI pores. These characteristics align closely with classic reports describing well-crystallized, low-defect MFI membranes where adsorption-driven diffusion dominates for condensable gases [52]. Similarly, membranes designed for efficient SF_6_ recovery have shown a permeance sequence of CO_2_ > H_2_ > He > N_2_ > CH_4_ > SF_6_, with the higher CO_2_ permeance reasonably explained by preferential adsorption and rapid diffusion within the zeolite framework [53].

Overall, the effort to upscale the membrane from capillary supports to conventional supports showed promising results. By applying the fundamental principle of enhanced heat transfer, we achieved rapid silicalite-1 membrane synthesis similar to the accelerated growth observed in the capillary support system. The improved heat transfer likely promoted a more uniform distribution across the support, as well as rapid heating of the synthesis solution and seeded support, enabling faster nucleation and crystal growth of silicalite-1. This approach demonstrates that the rapid synthesis strategy is not limited to capillary geometries but can be effectively extended to larger conventional supports, providing a viable pathway toward practical membrane fabrication and scale-up.

### 3.3. Comparison with Other Reports

**Figure 8:**
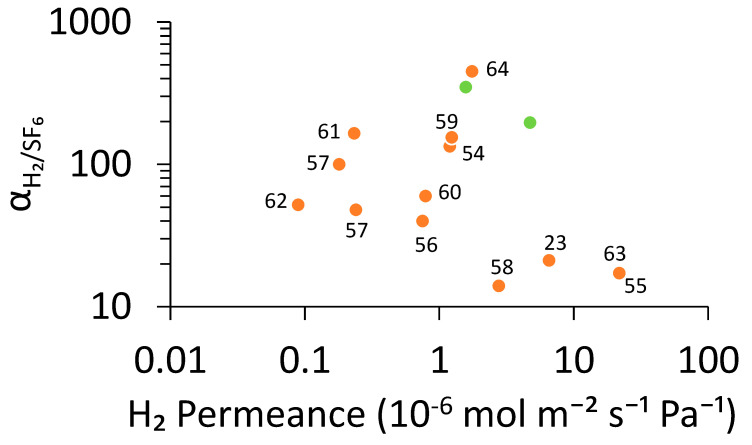
Relationship between separation factors and H_2_ permeance of MFI membranes. Silicalite-1 membranes on the conventional large-diameter support in this study (green) and previously reported membranes prepared by conventional heating method (orange) [23,54,55,56,57,58,59,60,61,62,63,64].

The performance of the silicalite-1 membranes synthesized in this study was compared with those reported in the literature. The 45 min membranes prepared in this work exhibited H_2_ permeance ranging from 1.6 × 10^−6^ to 4.7 × 10^−6^ mol m^−2^ s^−1^ Pa^−1^ and H_2_/SF_6_ ideal separation factor between 196 and 349. These values are comparable to or higher than several previously reported datasets obtained with much longer synthesis time (6–48 h), as shown in Figure S8. For instance, earlier studies, as shown in Figure 8, reported H_2_ permeance between 8.9 × 10^−8^ and 2.2 × 10^−5^ mol m^−2^ s^−1^ Pa^−1^, with corresponding H_2_/SF_6_ ideal separation factor varying widely from 14 to 450. The moderate permeance, combined with the relatively high ideal separation factor obtained in this study, indicates that our rapid synthesis method produced membranes with well-developed microporous structures and minimal defects. This suggests that efficient heat transfer in the newly designed reactor contributes to an improved membrane growth rate without compromising molecular sieving performance.

The N_2_/SF_6_ permeance ratio of 223 measured for the conventional support membrane in this study confirms its capability to discriminate between small (N_2_) and large (SF_6_) molecules. the same as the case of H_2_ over SF_6_ discussed earlier. The use of the N_2_/SF_6_ permeance ratio as a benchmark for membrane quality [65], serving as an indicator of defect density and microporous selectivity, has precedent in the literature; in the same report, the N_2_/SF_6_ value was 29. Coronas et al. reported N_2_/SF_6_ of ZSM-5 tubular membrane in the range of 140–300 [66], which is quite similar to our best value of 223. Another study reported an N_2_/SF_6_ separation factor of 325 for the SSZ-13 membrane, which was claimed for efficient SF_6_ recovery [53].

Considering the practical use of the membrane for SF_6_ recovery, polymeric membranes, particularly those made from commercial polysulfone (PSF) and polyimide, have been employed in N_2_/SF_6_ separation applications, demonstrating selectivity in the range of 20 to 40 and N_2_ permeance values 3.39 × 10^−10^–1.69 × 10^−9^ mol m^−2^ s^−1^ Pa^−1^ [53], and ideal selectivity of N_2_/SF was 14.4 and 16.3 at the feed pressure of 0.3 and 0.5 MPa, respectively [2]. In one study, the silicalite-1 membrane exhibited high H_2_ permeance (1.2 × 10^−6^ mol m^−2^ s^−1^ Pa^−1^) and excellent permselectivity for H_2_ over SF_6_ (α_H2/SF6_ = 134) at 298 K under a 0.1 MPa pressure drop, while α_N2/SF6_ = 38, with synthesis times ranging from 20 to 72 h [54]. Application of SSZ-13 zeolite membranes produced reported N_2_ permeance of 5.4 × 10^−8^ mol m^−2^ s^−1^ Pa^−1^, with ideal selectivity of 570, for N_2_/SF_6_, which far surpassed Knudsen selectivity and confirmed that this membrane shows promise for nitrogen removal from SF_6_ spent gas [53]. Zhang et al. claimed that the high N_2_ permeance (~1.7 × 10^−6^ mol m^−2^ s^−1^ Pa^−1^ in single-gas permeation) of silicalite-1 membrane synthesized via 17 h hydrothermal synthesis enabled efficient SF_6_ recovery from N_2_ despite a relatively low ideal separation factor (α_H2/SF6_ of ~55) [1]. Our silicalite-1 membrane exhibited better α_N2/SF6_ with comparable N_2_ permeance (1.0 × 10^−6^ mol·m^−2^·s^−1^·Pa^−1^), which is expected to be promising in the application of SF_6_ purification.

## 4. Conclusions

Rapid synthesis of silicalite-1 membranes was achieved through enhanced heat transfer using a small tubular support. Small seeds (~100 nm) provided high nucleation density and uniform intergrowth, producing defect-free membranes with excellent molecular sieving performance within 45 min of hydrothermal synthesis, whereas larger seeds (~1 µm) resulted in coarser, less uniform layers with variable selectivity. The reactor design with improved heat transfer in this study enabled uniform growth on conventional large-diameter supports, achieving high H_2_ permeance (4.7 × 10^−6^ mol m^−2^ s^−1^ Pa^−1^), high H_2_/SF_6_ ideal separation factor (up to 349), and an N_2_/SF_6_ ideal separation factor of 223, demonstrating the scalability of this approach without compromising membrane quality. These findings highlight that careful control of seed characteristics, synthesis parameters, and reactor configuration enables rapid and scalable fabrication of silicalite-1 membranes with superior gas separation performance, providing a foundation for advanced zeolite membrane applications.

## Figures and Tables

**Table 1:** Comparison of H_2_ permeance, SF_6_ permeance, and separation factors for membranes synthesized using conventional and capillary supports.

Support	Synthesis Time [min]	H_2_ Permeance [mol m^−2^ s^−1^ Pa^−1^]	SF_6_ Permeance [mol m^−2^ s^−1^ Pa^−11^]	α_H2/SF6_
**Conventional *^a^***	30	(2.4 ± 0.9) × 10^−6^	(4.4 ± 0.8) × 10^−8^	56 ± 20
	45	(2.6 ± 1.8) × 10^−6^	(1.3 ± 0.9) × 10^−8^	239 ± 92
**Capillary**	30	3.0 × 10^−7^	1.6 × 10^−9^	182
	45	4.9 × 10^−7^	2.0 × 10^−9^	244

*^a^* the mean and standard deviation of three membranes.

## Data Availability

The original contributions presented in the study are included in the article and supplementary material; further inquiries can be directed at the corresponding author.

## Supplementary Materials

The following supporting information can be downloaded at: https://www.mdpi.com/article/10.3390/membranes16030091/s1, Figure S1. SEM image of silicalite-1 seed crystals, L-seed: left, S-seed: right, scale bar represent 1 µm; Figure S2. XRD patterns of silicalite-1 seed crystals compared to IZA standard [37]; Figure S3. SEM image of the (a) cross-section and (b) surface of membranes synthesized using S-seed and 20 cm reactor. Scale bar: 5 µm; Figure S4. EDX-SEM cross-sectional analysis of membranes prepared using (a) L-seed and (b) S-seed. Si: red, Al: green, scale bar: 10 µm; Figure S5 Time dependence of separation factor and silicalite-1/Al_2_O_3_ peak ratio of silicalite-1 membranes on capillary supports using S-seed; Figure S6. SEM image of surface of seeded support rubbed with S-seed. Scale bar: 5 µm; Figure S7. SEM-EDX of the cross-section images of membranes synthesized in 30 and 45 min using S-seed on conventional support. Scale bar: 5 µm; Figure S8. Separation factors of MFI membranes with synthesis time. Silicalite-1 membranes on the conventional support in this study (green) and previously reported membranes which were prepared by conventional heating method (orange) [23,54,55,56,57,58,59,60,61,62,63,64].

## Abbreviations

The following abbreviations are used in this manuscript:

TPAOH	Tetra-*n*-propylammonium hydroxide
TPABr	Tetra-*n*-propylammonium bromide
TEOS	Tetraethyl orthosilicate
PTFE	Polytetrafluoroethylene
SEM	Scanning electron microscope
XRD	X-ray diffraction
XRF	X-ray fluorescence
EDX	Energy-dispersive X-ray
