# Supplementary material for: Versatile Use of the Small Tubular Reactor and Introduction of a Novel Design Reactor for Rapid Synthesis of Silicalite-1 Membranes
Source: Membranes (Basel). 2026 Mar 2;16(3):91. doi: 10.3390/membranes16030091 (PMC13027664; doi:10.3390/membranes16030091)
Supplement: Supplementary file 1 [file membranes-16-00091-s001.zip › membranes-4095037-supplementary.pdf]

Supplementary Information

Versatile Use of the Small Tubular Reactor and Introduction of a Novel Design Reactor for Rapid Synthesis of Silicalite-1 Membranes

Rizqan Jamal[1], Yuta Kayukawa[2], Ryouki Kitamura[3], Manabu Miyamoto[3], Yasuhisa Hasegawa[4] , Yasunori Oumi[5] , Shigeyuki Uemiya[3].*

[1]Department of Engineering Science, Graduate School of Engineering, Gifu University
[2]Department of Materials Science and Processing, Graduate School of Natural Science and Technology, Gifu University
[3]Department of Chemistry and Biomolecular Science, Faculty of Engineering, Gifu University, 1-1 Yanagido, Gifu, 501-1193, Japan
[4]The National Institute of Advanced Industrial Science and Technology
[5]Institute for Advanced Study, Gifu University

Table of Contents

1. Silicalite-1 seed crystals **2**

2. Rapid synthesis of silicalite-1 membrane on capillary supports **3**

3. Rapid preparation of silicalite-1 membrane on conventional support **5**

1. Silicalite-1 seed crystals

We successfully synthesized seed crystals of silicalite-1, which were confirmed to exhibit a similar structural pattern to the MFI pattern from The International Zeolite Association (IZA) standard[1], the XRD patterns can be seen in Fig S1. The crystals have an average size of approximately 1 µm, as shown in Fig. S2 and seed crystals contained 99.99% silica by XRF analysis.

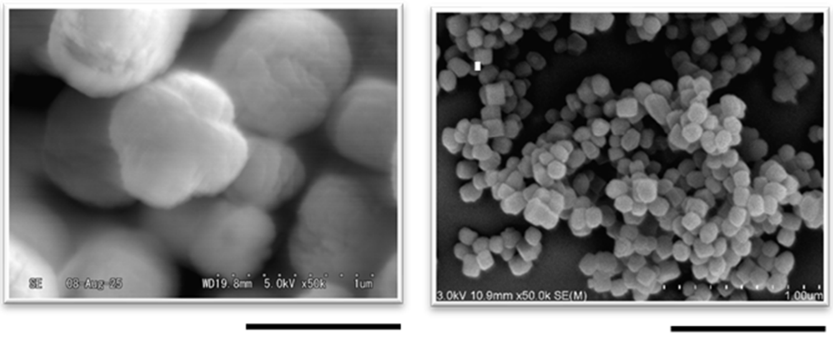

Figure S1. SEM image of silicalite-1 seed crystals, L-seed: left, S-seed: right.
Scale bar: 1 µm.

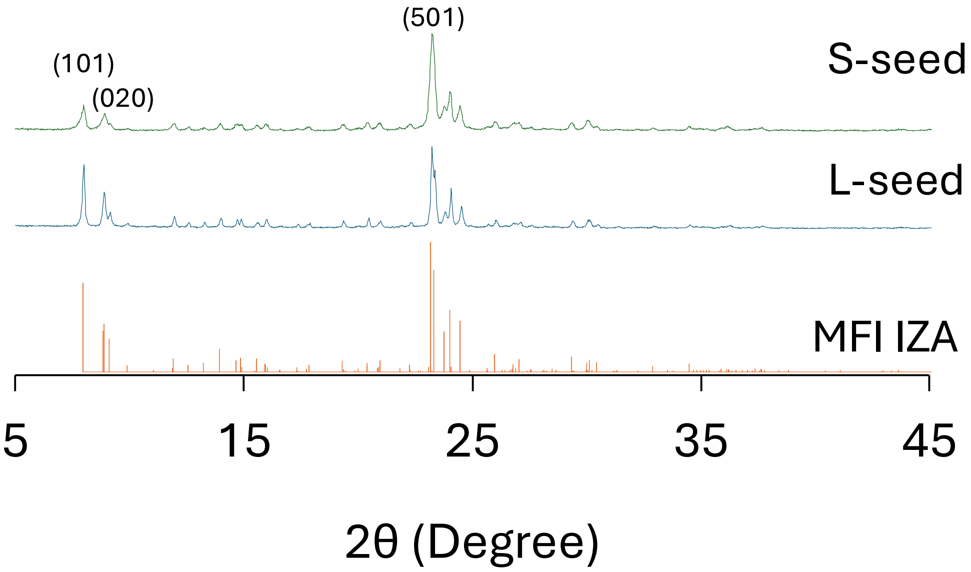

Figure S2. XRD patterns of silicalite-1 seed crystals compared to IZA standard[1].

2. Rapid synthesis of silicalite-1 membrane on capillary supports

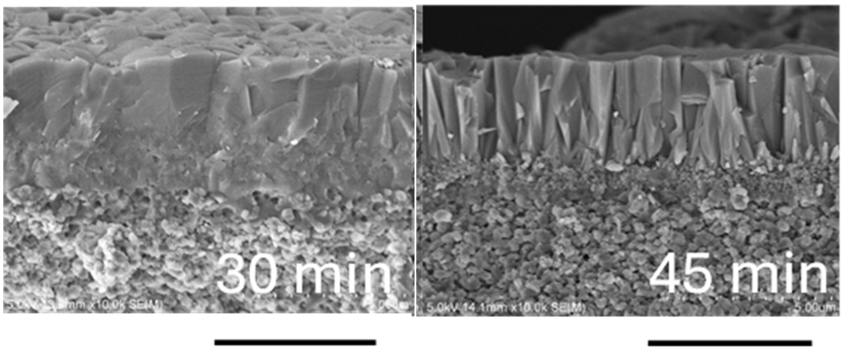

(a)

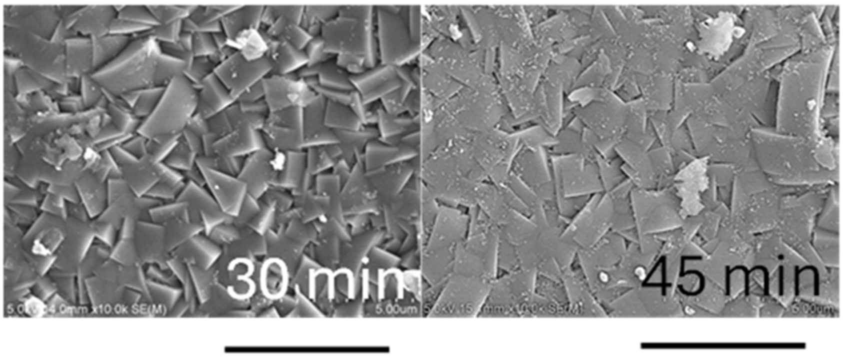

(b)

Figure S3. SEM images of the (a) cross-section and (b) surface of membranes synthesized on the capillary support using S-seed and 20 cm reactor. Scale bar: 5 µm.

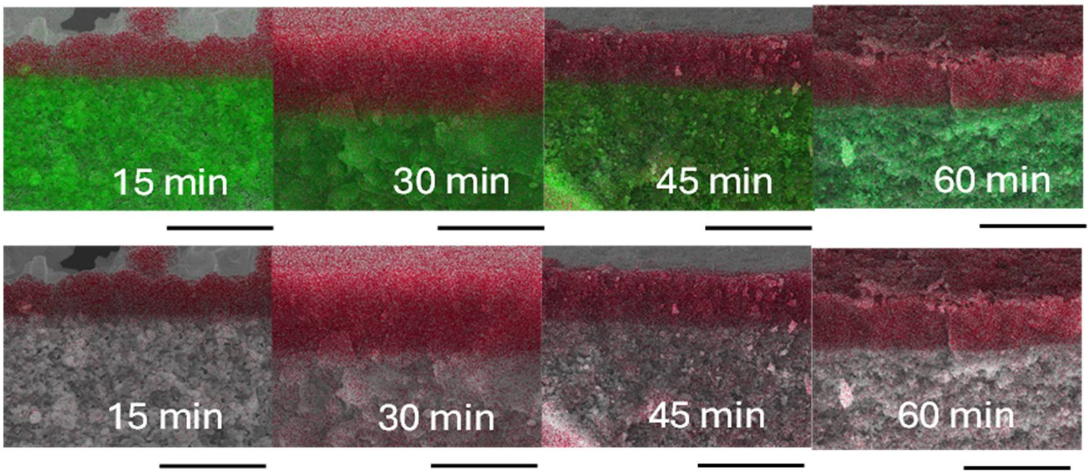

(a)

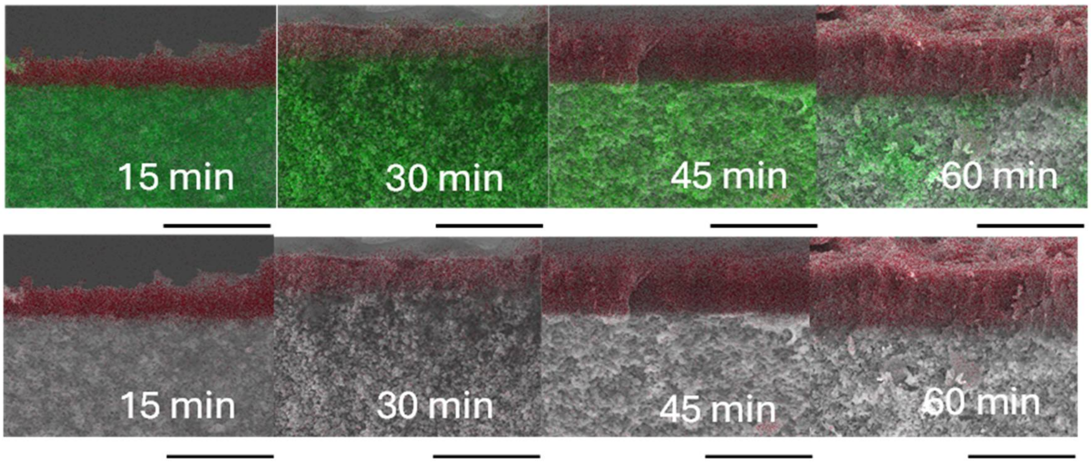

(b)

Figure S4. EDX-SEM cross-sectional analysis of membranes prepared on the capillary support using (a) L-seed and (b) S-seed. Si: red, Al: green, scale bar: 10 µm.

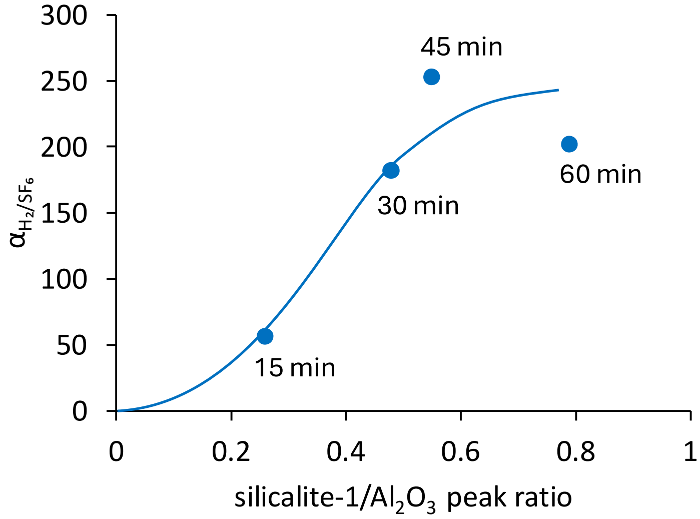

Fig. S5 Time dependence of separation factor and silicalite-1/Al_2O_3 peak ratio of silicalite-1 membranes on capillary supports using S-seed.

3. Rapid preparation of silicalite-1 membrane on conventional support

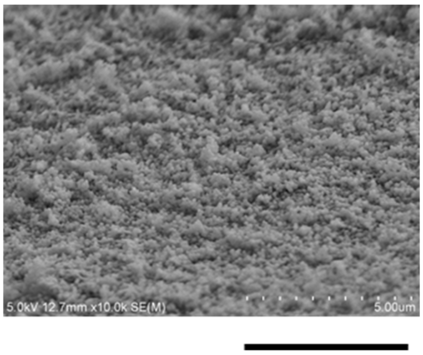

Figure S6**.** SEM image of surface of seeded support rubbed with S-seed. Scale bar: 5 µm.

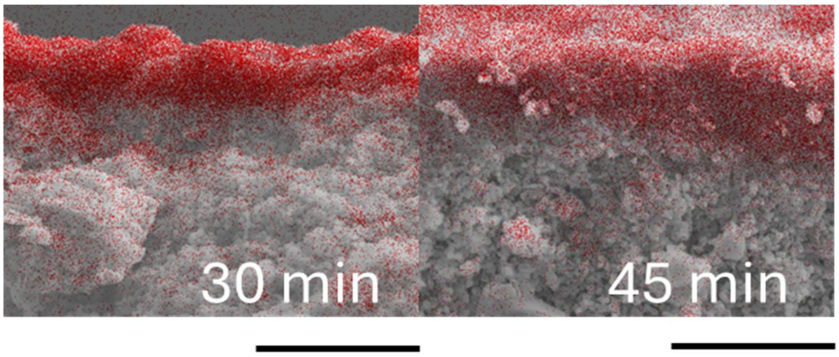

Figure S7. SEM-EDX of the cross-sectional analysis of membranes synthesized in 30 and 45 min using S-seed on conventional support. Scale bar: 5 µm.

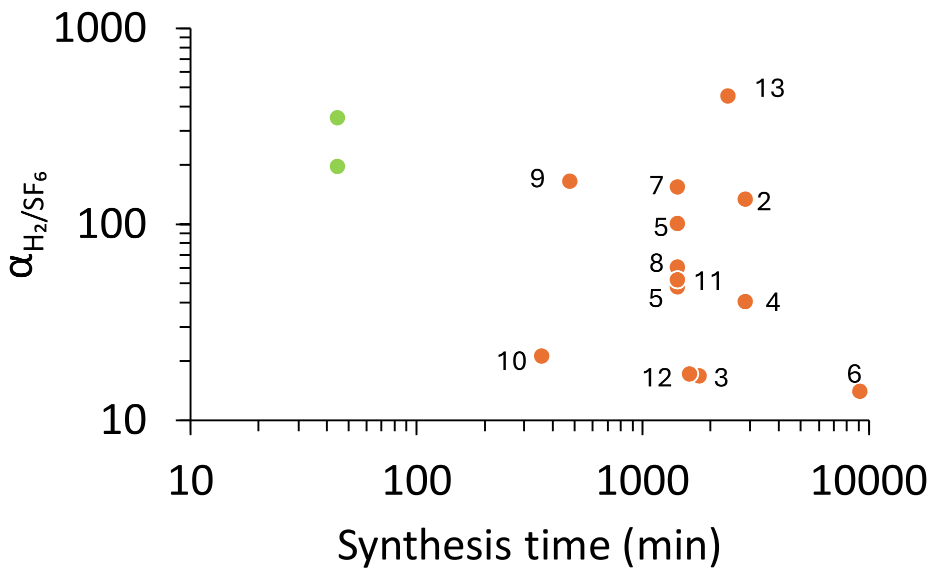

Figure S8. Relationship between Separation factors of MFI membranes with synthesis time. Silicalite-1 membranes on the conventional support in this study (green) and previously reported membranes prepared by conventional heating method (orange) [2–13].

References

1. Database of Zeolite Structures of The International Zeolite Association. Available online: https://america.iza-structure.org/IZA-SC/pow_plot_VerifSyn.php?ID=37 (accessed on 24 December 2025)

2. Xiao, W.; Yang, J.; Shen, D.; Lu, J.; Wang, J. Synthesis and Property of Silicalite-1 Membranes by Restricting Growth Method with Dilute Solution. *Microporous and Mesoporous Mater.* **2010**, *129*, 22–29, doi:10.1016/j.micromeso.2009.08.036.

3. Hedlund, J.; Sterte, J.; Anthonis, M.; Bons, A.J.; Carstensen, B.; Corcoran, N.; Cox, D.; Deckman, H.; De Gijnst, W.; De Moor, P.P.; et al. High-Flux MFI Membranes. *Microporous Mesoporous Mater.* **2002**, *52*, 179–189, doi:10.1016/S1387-1811(02)00316-5.

4. Mabande, G.T.P.; Pradhan, G.; Schwieger, W.; Hanebuth, M.; Dittmeyer, R.; Selvam, T.; Zampieri, A.; Baser, H.; Herrmann, R. A. Study of Silicalite-1 and Al-ZSM-5 Membrane Synthesis on Stainless Steel Supports. *Microporous Mesoporous Mater.* **2004**, *75*, 209–220, doi:10.1016/J.MICROMESO.2004.07.009.

5. Bonhomme, F.; Welk, M.E.; Nenoff, T.M. CO_2 Selectivity and Lifetimes of High Silica ZSM-5 Membranes. *Microporous Mesoporous Mater.* **2003**, *66*, 181–188, doi:10.1016/j.micromeso.2003.09.005.

6. Algieri, C.; Golemme, G.; Kallus, S.; Ramsay, J.D.F. Preparation of Thin Supported MFI Membranes by in Situ Nucleation and Secondary Growth. *Microporous Mesoporous Mater.* **2001**, *47*, 127–134, doi:10.1016/S1387-1811(01)00393-6.

7. Xiao, W.; Yang, J.; Lu, J.; Wang, J. A Novel Method to Synthesize High Performance Silicalite-1 Membrane. *Sep. Purif. Technol.* **2009**, *67*, 58–63, doi:10.1016/j.seppur.2009.03.007.

8. Mabande, G.T.P.; Schwieger, W.; Hanebuth, M.; Dittmeyer, R.; Selvam, T. Silicalite-1 Membranes Prepared via One-Step In Situ Seeding. *Stud. Surf. Sci. Catal.* **2004**, *154*, 695–702, doi:10.1016/S0167-2991(04)80872-3.

9. Dong, J.; Lin, Y.S.; Hu, M.Z.C.; Peascoe, R.A.; Payzant, E.A. Template-Removal-Associated Microstructural Development of Porous-Ceramic-Supported MFI Zeolite Membranes. *Microporous Mesoporous Mater.* **2000**, *34*, 241–253, doi:10.1016/S1387-1811(99)00175-4.

10. Qiu, H.; Xu, N.; Kong, L.; Zhang, Y.; Kong, X.; Wang, M.; Tang, X.; Meng, D.; Zhang, Y. Fast Synthesis of Thin Silicalite-1 Zeolite Membranes at Low Temperature. *J. Membr. Sci.* **2020**, *611*, 118361, doi:10.1016/j.memsci.2020.118361.

11. Noack, M.; Mabande, G.T.P.; Caro, J.; Georgi, G.; Schwieger, W.; Kölsch, P.; Avhale, A. Influence of Si/Al Ratio, Pre-Treatment and Measurement Conditions on Permeation Properties of MFI Membranes on Metallic and Ceramic Supports. *Microporous Mesoporous Mater.* **2005**, *82*, 147–157, doi:10.1016/j.micromeso.2005.03.007.

12. Sandström, L.; Lindmark, J.; Hedlund, J. Separation of Methanol and Ethanol from Synthesis Gas Using MFI Membranes. *J. Membr. Sci.* **2010**, *360*, 265–275, doi:10.1016/j.memsci.2010.05.022.

13. Tanizume, S.; Yoshimura, T.; Ishii, K.; Nomura, M. Control of Sequential MTO Reactions through an MFI-Type Zeolite Membrane Contactor. *Membranes* **2020**, *10*, 26, doi:10.3390/membranes10020026.
